# Tree‐Cavity Denning of Güiña (*Leopardus guigna*) and Breeding Productivity From Camera Trap Records

**DOI:** 10.1002/ece3.71723

**Published:** 2025-09-06

**Authors:** Fernando J. Novoa, Mariana Ayala, José Infante‐Varela, José Tomás Ibarra, Jesús Díaz, Tomás A. Altamirano, Nicolás Gálvez

**Affiliations:** ^1^ Programa de Doctorado en Ciencias Antárticas y Subantárticas & Cape Horn International Center for Global Change Studies and Biocultural Conservation (CHIC) Universidad de Magallanes Punta Arenas Chile; ^2^ ECOS (Ecosystem‐Complexity‐Society) Co‐Laboratory, Austral Mountain Conservation and Research (CIMA Lab) & Wildlife Ecology and Coexistence Lab, Center for Local Development (CEDEL) Pontificia Universidad Católica de Chile (PUC) Villarrica Chile; ^3^ Programa de Doctorado en Ecosistemas Forestales y Recursos Naturales & Laboratorio de Fauna Silvestre Instituto de Conservación, Biodiversidad y Territorio, Facultad de Ciencias Forestales y Recursos Naturales, Universidad Austral de Chile Valdivia Chile; ^4^ Faculty of Agriculture and Natural Systems, Center of Applied Ecology and Sustainability (CAPES) & Center for Intercultural and Indigenous Research (CIIR) Pontificia Universidad Católica de Chile (PUC) Santiago Chile; ^5^ Fundación Mar Adentro Pucón Chile

**Keywords:** feline, forests, Kod kod, natural history, remnant tree

## Abstract

Tree cavities are critical habitats for numerous vertebrate species, serving as keystone resources for nesting, roosting, and shelter. We document the first evidence of an individual güiña (*Leopardus guigna*) breeding within a tree cavity of a standing dead tree. We explore its implications on breeding productivity and complementing this record with evidence from camera trap surveys conducted in temperate forests of south‐central Chile. These findings enhance our understanding of the species' natural history, breeding behavior, and habitat preferences. Additionally, we discuss the conservation implications of this discovery, offering new insights into the breeding ecology and habitat selection of one of South America's most elusive felines.

## Graphical abstract

Tree cavities are critical habitats for numerous vertebrate species. We report the first evidence of güiña breeding in a tree cavity, with insights from camera trap surveys in south‐central Chile's temperate forests. These findings enhance our understanding of the species' natural history, breeding behavior, and habitat preferences.

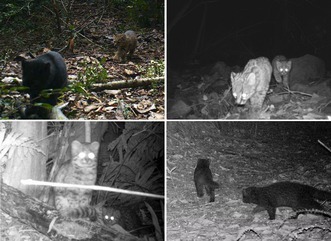

## 1 Introduction

The güiña or kod kod cat ( *Leopardus guigna* ) is the smallest wild cat in the Americas (ca. 2 kg) with the most restricted distribution in the world (Napolitano et al. 2015). The güiña is an elusive and cryptic species endemic to the forested Mediterranean and temperate rainforest biomes of Chile and Argentina, and is patchily distributed in a narrow range over latitudes 30°–50° S (Napolitano et al. 2015). In Chile, in its southern range, the species is strongly associated with moist temperate mixed forests of the southern Andean and Coastal ranges, particularly the Valdivian and Araucaria forests of Chile, which are characterized by abundant Southern Beech trees (*Nothofagus* spp.) and an understory dominated by bamboo species (Nowell and Jackson 1996; Lucherini et al. 2000).

The güiña is categorized as Vulnerable, with a decreasing population trend, on the IUCN Red List, due to habitat loss and fragmentation from human population growth and deforestation in Chile's temperate rainforests (Napolitano et al. 2015). Preference for native forest (Acosta‐Jamett and Simonetti 2004) makes the güiña sensitive to landscape change (Gálvez et al. 2013); although recent studies show the ability to survive in highly modified agricultural landscapes (Gálvez et al. 2018, Gálvez, Infante, et al. 2021). Forest cover is a critical ecological requirement for the species, influencing its spatial dynamics. Whether continuous or in agricultural mosaics, it provides essential habitat refugia, offering suitable areas for shelter and movement (Gálvez et al. 2013; Fleschutz et al. 2016).

It is assumed that the species “builds nests” on top of trees or in dense clumps of bamboo and it is stated that it can have between 1 and 4 offspring, although both characteristics are not supported by any study (Quintana et al. 2009). Breeding productivity is defined as the number of kittens/juveniles per reproductive female, defined as the females observed in the presence of offspring (Smith and Smith 2007; Primack et al. 2001). This information is vital, particularly given the high levels of forest loss affecting the species' habitat. However, breeding behavior, denning sites, and habitat attributes remain poorly understood. A probable mating season has been suggested to occur in early spring (August–September; Peckham 2023), based on size and dentition, collected a juveniles male güiña (Dunstone et al. 2002; Freer 2004).

Tree cavities are generally considered critical habitat for a range of species, from invertebrates through birds to mammals, because they serve as a keystone resource, providing nesting, roosting, and sheltering sites for numerous vertebrate species (Altamirano, Ibarra, Martin, and Bonacic 2017; Bonaparte et al. 2024). Remaining forest stands showing suitable habitat for cavity‐using wildlife, such as sites with large‐decaying trees and standing dead trees (i.e., snags), are commonly being degraded (Vergara and Armesto 2009; Ibarra and Martin 2015). Snags are frequently considered a keystone habitat attribute because of the disproportionate number of species they support in relation to young healthy trees (Ibarra et al. 2020). Although they play a crucial ecological role, there is limited knowledge about how species like the güiña rely on snags for essential behaviors such as reproduction, especially in forest landscapes experiencing degradation.

We document the first evidence of güiña breeding within a tree cavity of a snag in temperate forests. We explore the implications of this finding for breeding productivity and complement this finding with evidence from camera trap surveys. We also discuss the significance of this discovery for the species and its potential implications for conservation. Our study provides novel insights into the breeding behavior and habitat selection of one of South America's most elusive felines.

## 2 Methods

### 2.1 Study Area

The study was conducted in four regions of South‐Central Chile: El Maule, La Araucanía, Los Ríos, and Los Lagos. These regions encompass both Mediterranean and temperate macro bioclimates. El Maule, located mainly in central Chile (23°–39° S), has a Mediterranean macro bioclimate characterized by scrubland and sclerophyll forests. The vegetation includes interior Mediterranean communities such as Espinal ( *Acacia caven* ), Litre (*Lithraea caustica*), Boldo ( *Peumus boldus* ), and Corcolen (*Azara integrifolia*) along with diverse grasslands composed of native and exotic species (Luebert and Pliscoff 2017). In contrast, La Araucanía, Los Ríos, and Los Lagos have a temperate macro bioclimate that extends in southern Chile (37°–55° S). Vegetation in these regions includes early successional stages of forests at lowlands dominated by broadleaf species such as Roble beech ( *Nothofagus obliqua* ), Coigüe ( *Nothofagus dombeyi* ), Ulmo ( *Eucryphia cordifolia* ), and Chilean laurel (*Laurelia sempervirens*). The understory was mostly dominated by bamboo species (*Chusquea* spp.), *Rhaphithamnus spinosus*, different species of *Azara* and *Berberis*, and tree saplings (see also Altamirano et al. 2013; Altamirano, Ibarra, Martin, and Bonacic 2017; Luebert and Pliscoff 2017).

### 2.2 Reproductive Monitoring

**FIGURE 1:**
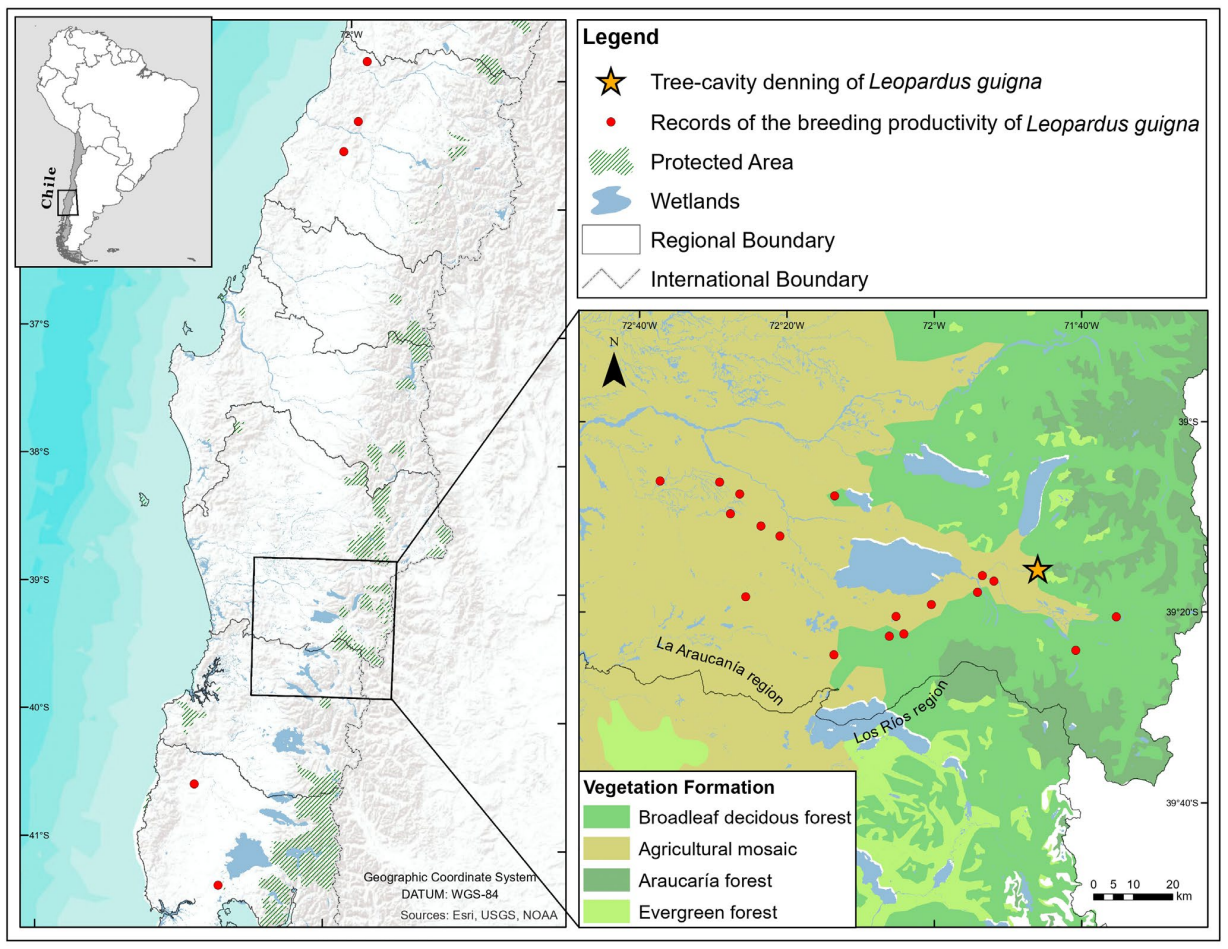
Study area of camera‐trap surveys conducted in the Maule, La Araucanía, Los Ríos, and Los Lagos regions of south‐central Chile. Red dots indicate cameras that recorded an adult *Leopardus guigna* with a kitten/cub.

For the nestweb long‐term project in Chilean temperate rainforests of La Araucania region, during 15 breeding seasons (2008–2023), we searched for nests of cavity‐nesting vertebrates and checked the interior of tree cavities used for nesting. We checked the cavities using a wireless monitoring system with a telescopic pole that reached up to 15 m high (see Altamirano, Ibarra, Martin, and Bonacic 2017). After detecting the species with a cub in the cavity, we deployed one camera‐trap (Bushnell model 16MP Trophy Cam HD Essential E3) in front of a tree cavity in a standing dead Roble beech ( *Nothofagus obliqua* ) tree (39°15′25.4″ S, 71°45′57.3″ W; Figure 1). This camera was located approximately 4 m above ground. The distance between the camera and tree cavity was approximately 2 m. Camera triggers were set to high sensitivity, three pictures per trigger and to record videos for 10–30 s with rapidfire intervals, and no delay between triggers.

### 2.3 Habitat Attributes

After the breeding season, we assessed site characteristics at three different scales: (a) cavity‐scale: origin (i.e., excavated or formed through natural decay processes), we measured the height using a tree‐climbing leader and a measuring tape (measured from the ground to the lower edge of the cavity entrance). Additionally, we recorded the entrance orientation, cavity entrance width and height, vertical and horizontal depth; (b) tree‐scale: tree species, diameter at breast height (DBH), diameter at cavity height (DCH), coverage by vines and epiphytes, level of decay (decay classes: 1 = live, healthy tree; 2a = live tree with sign of wood‐boring arthropods and/or fungal decay; 2b = nearly dead tree with broken top and advanced levels of decay, with 20% live branches; 3 = standing dead tree in progressive states of decay; adapted from Thomas et al. 1979); (c) stand‐scale: habitat type (e.g., Second‐growth forest = 35–100 years old), canopy cover, understory vegetation coverage, tree density (only trees with DBH > 12.5 cm), volume of coarse woody debris (with a diameter of ≥ 7.5 cm), and signs of recent human activities (e.g., logging, grazing, or fire; for a review see Ibarra et al. 2014; Altamirano, Ibarra, Martin, and Bonacic 2017).

### 2.4 Güiña Parental Activity in Cavity

To assess the parental activity of the güiña, we used time‐stamped data from images captured by camera trap (Chaudhary et al. 2020). By organizing all detection events according to the time of day, we calculated hourly detection frequencies to estimate the güiña's activity pattern over a 24‐h period. Activity patterns were estimated nonparametrically using kernel density functions (Ridout and Linkie 2009). For the general activity pattern analysis, we used the complete dataset of photographic records related to tree‐cavity denning behavior. Each event was classified as either a cavity entrance or exit by an adult güiña, allowing us to compare the temporal activity patterns associated with these two behaviors. After the activity pattern had been generated for each behavior, the behaviors were compared, and the overlap coefficient (Δ) was estimated (Ridout and Linkie 2009). This coefficient ranged from 0 (no overlap) to 1 (complete overlap) and was obtained by taking the minimum of the density functions of the two cycles being compared at each time point (Monterroso et al. 2014).

We processed the images captured by camera trap using the EcoAssist (van Lunteren 2023) and classified them by species activity with Timelapse2 (Greenberg et al. 2019), incorporating the MegaDetector learning model (Machine Learning for Wildlife Image Classification). This model automatically identifies the presence of animals in images. To ensure maximum sensitivity, we applied the most conservative detection confidence threshold of 0.01 in EcoAssist, a threshold previously validated with a 100% success rate (i.e., all animals present in the images were accurately detected). After this automated detection process, an observer conducted a manual review of the images to verify the results.

### 2.5 Güiña Breeding Productivity in Camera Trap Surveys

Camera traps were installed to detect individuals moving on the forest floor across different vegetation formations (native forests, shrublands, and commercial forest plantations). Cameras were tied up against trees or rocks at a height of 15–30 cm above the ground. While we are detecting individuals, we are not identifying them at the individual level but at the species level. We collected records of güiña females with their kitten/cub or juveniles from camera trap surveys in El Maule, La Araucanía, Los Ríos, and Los Lagos Regions (Figure 1). Cameras were deployed at 437 unique locations, with one to five sampling seasons per location, between 2012 and 2024. Cameras operated from one to two months per season, and total sampling effort was 51,610 camera days. The location of sampling units was randomly selected, whether randomizing cells in a grid overlaid on the study areas (180 cameras in La Araucanía, see Gálvez et al. 2018, Gálvez, Meniconi, et al. 2021 for details; 90 cameras in Los Ríos and 90 cameras in Los Lagos) or randomizing exact camera locations, setting a minimal distance of 2 km between cameras and constraining selection to natural vegetation formations inside commercial forest plantations (40 cameras in El Maule region and 40 cameras in Los Lagos Region). Both design types used the home range size of güiña for determining cell size or distance between cameras (Johnson and Franklin 1994; Kasper et al. 2012; Schüttler et al. 2017). Camera models used were either Bushnell Trophy Cam HD Aggressor or Essential E3 and were programmed to take three photographs at each animal detection or 15‐s videos. All güiña records were reviewed in DigiKam or Timelapse programs for obtaining sequences with kittens or juveniles, where we noted the number of individuals in the sequence. We report the total independent records of güiña, which correspond to those with more than 60 min difference from the previous record in the same camera, and the observed breeding productivity, which corresponds to the average kittens or juveniles per female observed in a sequence (Monterrubio‐Rico et al. 2024). We assumed that adults with kittens/cubs or juveniles were female, as male felines do not raise their offspring.

## 3 Results

### 3.1 Reproductive Monitoring

**FIGURE 2:**
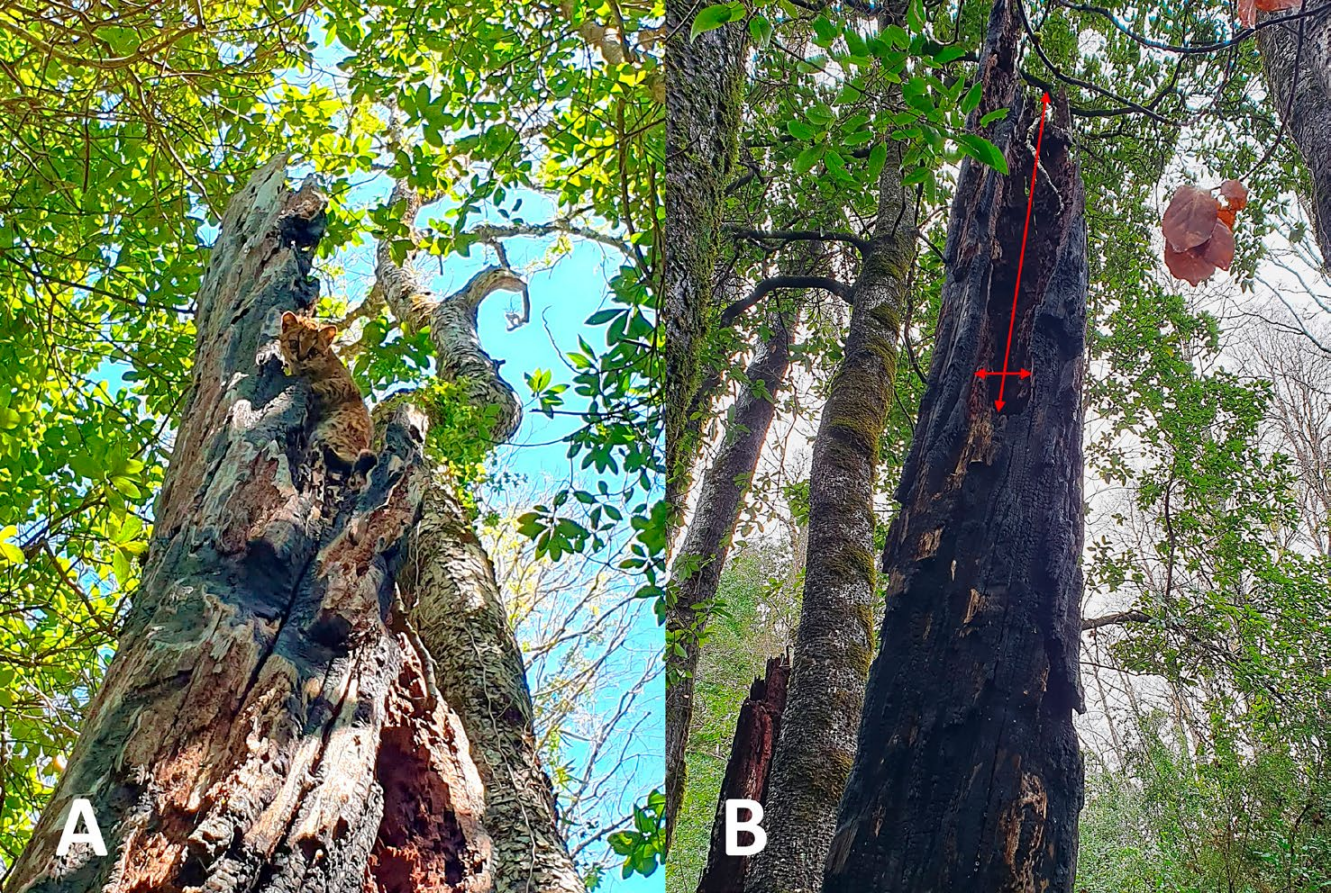
An adult of *Leopardus guigna* at the moment the tree cavity denning was first recorded (A). Tree cavity denning within a natural cavity of *Nothofagus obliqua* located in a second‐growth forest, exhibiting signs of fire damage. The red arrows indicate the dimensions of the cavity entrance (B).

On 24 October 2023 at 15:30 h, we recorded an adult güiña with one newborn kitten/cub (i.e., few days old) inside the tree cavity of a snag (Figure 2A,B). The kitten/cub was just opening its eyes at the time of the first encounter. In total, 1793 videos and 5405 images were recorded over 65 camera trap nights in the güiña tree cavity den (October 24, 2023, to December 28, 2023). We captured images of an adult individual and one kitten/cub using the tree cavity as den continuously for 33 days. According to the observed pelage pattern and number of adult güiñas in front of the camera, we presume that only one adult individual visited the cavity. Photographic captures from the adult and kitten/cub were taken both during the day and night. The female visually showed a low body condition score possibly due to nursing the kitten/cub.

### 3.2 Habitat Attributes

The cavity used as a den was inside a standing dead Roble beech tree (Figure 2A). The cavity was produced by fire and decay processes (Figure 2B). The cavity had been used for nesting by a Chimango Caracara ( *Milvago chimango* ) 12 years ago and by an Austral Thrush ( *Turdus falcklandii* ) 5 years ago. The cavity did not contain any material (e.g., leaves, bark, fur) while it was being used by the güiña. The Roble beech tree was a remnant tree or habitat legacy located in a second‐growth forest in lowland forests dominated by broadleaf species in the La Araucanía region, along with other medium‐sized trees and several snags (Table 1).

**TABLE 1:** Characteristics of cavity, tree, and stand used by *Leopardus guigna* in temperate forests, Chile.

Tree cavity dening variable	
**Cavity**	
Origin	Non‐excavated
Type	Chimney
Cavity height (m)	3.3
Entrance orientation (°)	330
Entrance width (cm)	15.2
Entrance height (cm)	—
Vertical cavity depth (cm)	30
Horizontal cavity depth (cm)	25
**Tree**	
Species	*Nothofagus obliqua*
Diameter at cavity height (cm)	55
Diameter at breast height (cm)	63
Vine and epiphyte cover (%)	0
Decay class	3
**Stand**	
Habitat type	Second‐growth forest
Canopy cover (%)	90
Understory cover (%)	35
Tree density (no./ha)	761.6
Coarse woody debris (m^3^/ha)	38,775
Coarse woody debris (%)	5
Signs of recent human disturbance	Fire

*Note:* —, No data could be collected.

### 3.3 Güiña Parental Activity in Cavity

**FIGURE 3:**
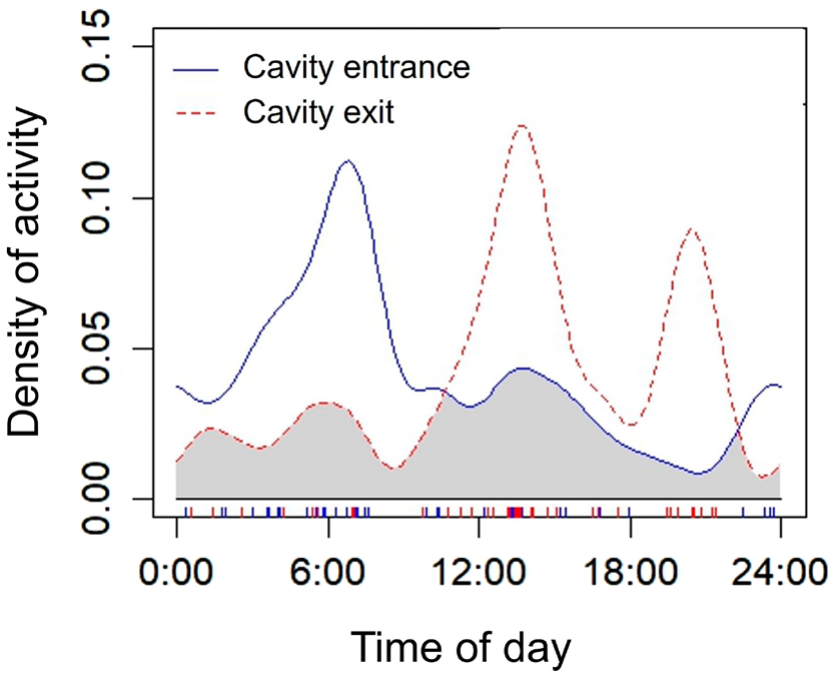
Estimated activity patterns of cavity use by an adult *Leopardus guigna* . The overlap of activity density curves is shown for each comparison. Independent records are indicated by tick marks along the *x*‐axis of the plot.

**FIGURE 4:**
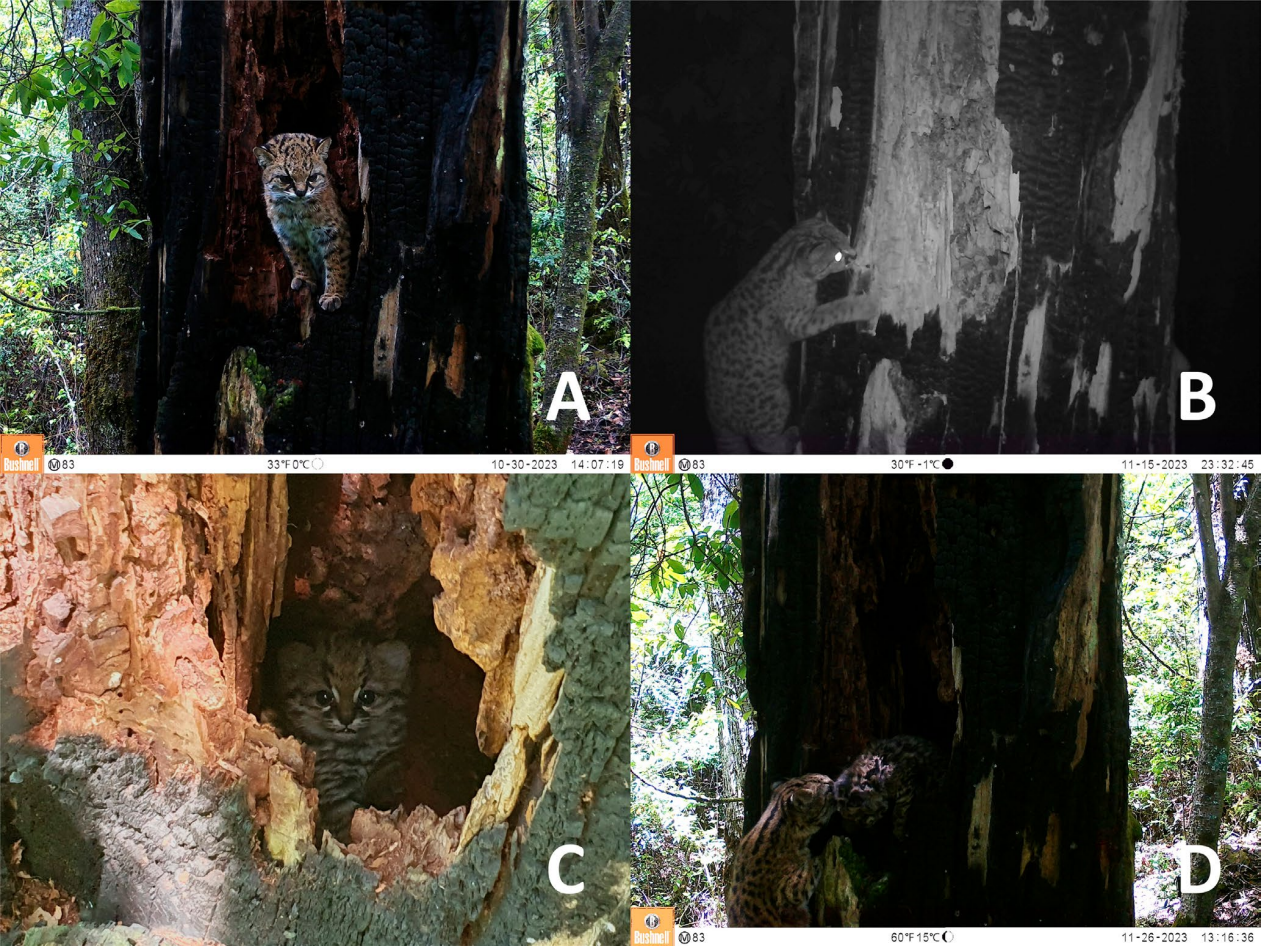
Records of an adult and a kitten/cub of *Leopardus guigna* using a cavity in a dead standing tree in the Chilean temperate forests. Adult exiting the cavity during the afternoon (A). Adult entering the cavity during the night (B). Kitten/cub over one month old inside the cavity (C). Adult carrying the kitten/cub to leave the cavity (D).

During the entire recording period, the adult güiña was observed entering and exiting the cavity without prey, indicating that she was likely solely feeding milk to the kitten/cub. The adult frequently entered and exited the cavity, presumably to feed the kitten/cub. In total, we recorded 42 events of the adult leaving the cavity followed by its subsequent return. We recorded two concentrations of adult activity outside the cavity (Figure 3). One concentration of activity occurred at midday (12 pm to 2 pm; Figures 3 and 4A), and the adult did not return to the cavity until dusk (11 pm to 12 am; Figure 4B) or the early morning of the following day (5 am to 7 am). Another concentration of activity occurred at sunset (8–9 pm), and the adult did not return to the cavity until dusk (4–7 am) or midday the next day (12–1 pm). The overlap in activity patterns between cavity entrance and exit behaviors was moderate, with an estimated overlap of 50%. On average, the adult spent 9 h outside the cavity per day. The longest period the adult remained outside the cavity was 23 h. The first time the kitten/cub was observed peeking out of the cavity was on November 1, 2023. The frequency of kitten/cub activity around the cavity entrance increased as it grew (Figure 4C). The kitten/cub and the adult left the cavity after 33 days from first detection on November 26, 2023, at 1:16 pm (Figure 4D). The adult removed the kitten/cub from the cavity, and they did not return to use it afterward. We estimate that the kitten/cub was one and a half months old at the time it left the cavity.

### 3.4 Güiña Breeding Productivity in Camera Trap Surveys

**FIGURE 5:**
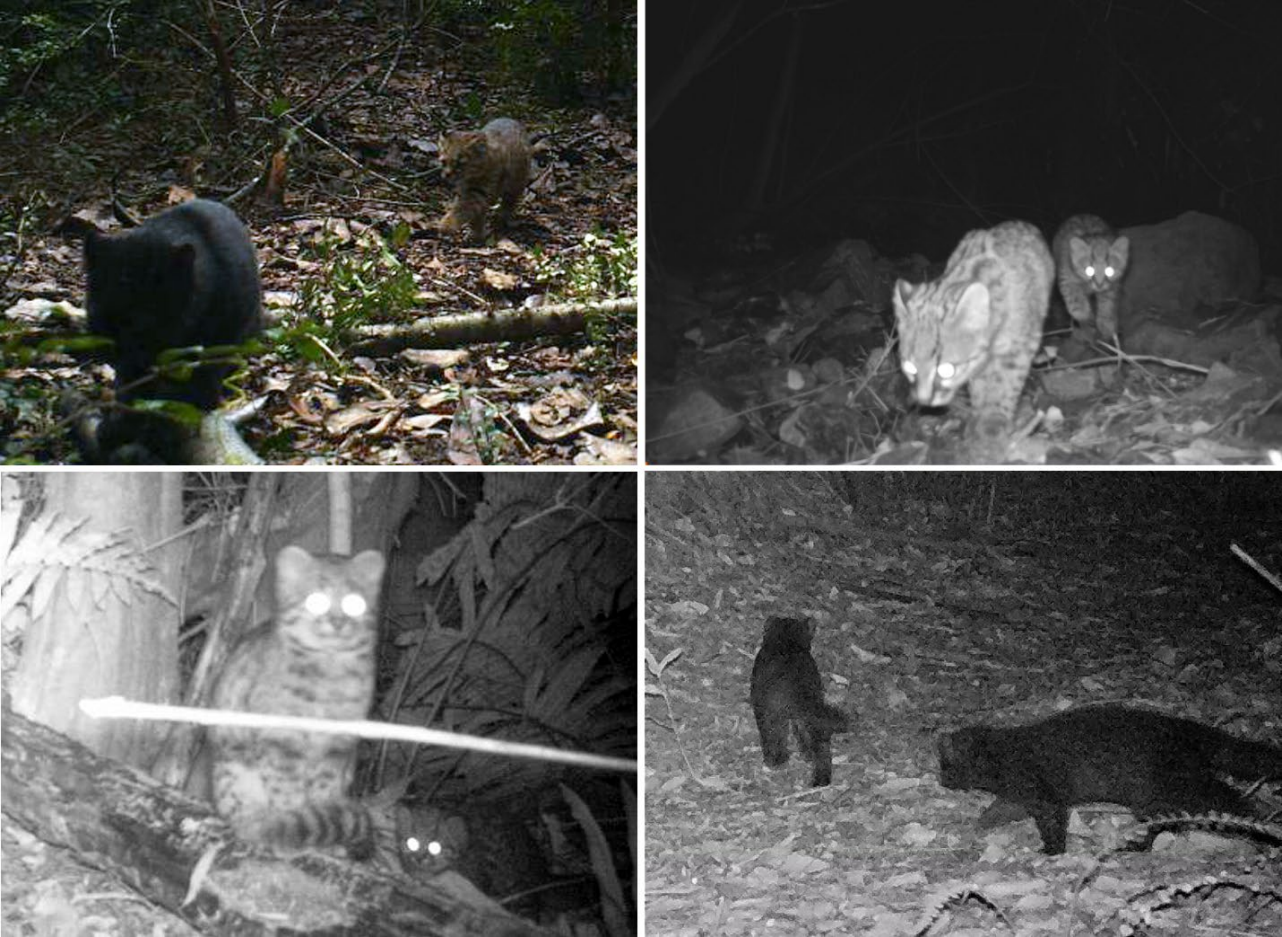
Records of an adult and a kitten/cub or juvenile *Leopardus guigna* captured by camera traps set up in south‐central Chile.

In our camera trap surveys, we obtained a total of 1830 independent detections of güiña. The detection of females with young individuals corresponds to ~1.6% of the total number of detections (*n* = 29). Güiña cubs were detected in 24 unique locations, with three sites yielding more than one independent detection. Three mother–cub pairs were recorded in sclerophyllous forests within pine plantations in the Maule Region. In the La Araucanía Region, at least 19 detections—plus five potential pseudoreplicates—were observed within a mosaic of temperate forests and agricultural landscapes. In Los Lagos, one detection occurred in a temperate forest within pine plantations and another in temperate forests within agricultural land (Figure 1). No females were observed accompanied by more than one cub (Figure 5). Kittens/cubs and juveniles were observed in all seasons of the year. In terms of color morph, 24.1% of the detections of the accompanying parent corresponded to melanistic specimens, while 10.3% of the total juvenile detections were melanistic.

## 4 Discussion

We recorded the first photographic evidence of güiña denning inside a tree cavity, located in a standing dead tree in a second‐growth forest stand. Additionally, we provide the first estimate of the species' breeding productivity based on extensive camera trap surveys. Our findings expand our understanding of the güiña's natural history, reproductive behavior, and life‐history traits.

The use of cavities in standing dead trees or snags as denning sites suggests that scattered large‐decaying trees and snags may be important for the reproduction of güiña. Although little is known about its reproductive behavior, it has been suggested güiñas give birth in constructed “nests” located in trees or on dense clumps of bamboo (Quintana et al. 2009). In contrast, we observed that the cavity contained no material, and the güiña showed no signs of attempting to construct a nest but rather used the available cavity.

Previous camera trap studies in southern Chile have recorded guiñas using living trees (Moreira‐Arce et al. 2021) and documented the use of tree cavities by guiñas as shelter sites (Vergara et al. 2024) and hunting sites (Altamirano et al. 2013). Therefore, this record expands that the güiña may use tree cavities as denning sites. Denning sites are unknown for most wild felids. Ocelots ( *Leopardus pardalis* ) have been described to locate their den near or under dense thornshrub cover, but also use dense grass growth for this purpose (Laack et al. 2005). Scattered large‐decaying trees and snags can be considered keystone structures for vertebrates, and they play a crucial role in maintaining the population persistence of cavity‐nesting species (Le Roux et al. 2018; Prevedello et al. 2018). In Andean temperate forests, 29 bird species, six mammals, three reptiles, and one amphibian have been previously described using cavities during their life cycle (Ibarra et al. 2014, 2020; Altamirano, Ibarra, Martin, and Bonacic 2017; Altamirano, Ibarra, Novoa, et al. 2017; Novoa et al. 2019). Snags are often seen as wasteful or unhealthy by the public and forest managers, leading to their removal (Ibarra and Martin 2015). However, they provide critical habitats for various species, preventing local extinctions in second‐growth forests (Altamirano, Ibarra, Martin, and Bonacic 2017; Ibarra et al. 2020). In these forests, snags, although comprising only 15% of cavity trees, contain more cavities than live trees and are important for cavity‐nesting vertebrates (Ibarra et al. 2020).

The presence of large‐decaying trees and snags, large cavities, and high canopy cover may influence güiña's habitat selection, providing shelter and food availability for its kitten/cub. An elevated den (at 3.3 m above ground in this case) may offer protection against terrestrial predators like foxes (i.e., *Lycalopex* sp.), puma ( *Puma concolor* ), and domestic dogs (Silva‐Rodríguez et al. 2007; Monteverde and D'Oliveira 2010; de Oliveira and Pereira 2013; Hunter 2015; Peckham 2023).

The area where the güiña's den was found had been affected by a fire over 80 years ago, which resulted in the formation of a second‐growth forest. Additionally, reproductive records from our extensive camera trap surveys indicate the species is capable of breeding within human‐dominated and altered landscapes. Although güiñas have previously been recorded in exotic pine and eucalyptus plantations, fragmented landscapes, and on the fringes of rural settlements and agricultural areas (Sanderson et al. 2002; Acosta‐Jamett and Simonetti 2004), reproductive behavior had not been documented in these ecosystems until now. It has been suggested that güiña can tolerate over 80% of habitat loss within a home range (Gálvez et al. 2018). Access to well‐developed understory or native forest patches, with the presence of prey, may allow the species to persist in altered habitats (Napolitano et al. 2015; Sanderson et al. 2002; Gálvez et al. 2013; García et al. 2021).

The adult güiña concentrated its activity in the cavity at sunset and midday, similar to patterns observed in southern Chile (Sanderson et al. 2002; Hernández Muñoz 2010; Delibes‐Mateos et al. 2014). Previous studies also noted crepuscular activity (Sanderson et al. 2002; Freer 2004). Güiñas show arrhythmic activity, with equal activity levels day and night, increasing on dry days (Dunstone et al. 2002; Peckham 2023). This could be a way to minimize thermoregulatory costs in cold, wet rainforests (Dunstone et al. 2002). Small mammal densities are lowest in spring (Muñoz‐Pedreros 1992; Freer 2004), coinciding with the study period, suggesting güiñas track diurnal prey to compensate (Delibes‐Mateos et al. 2014). Figueroa et al. (2018) noted higher consumption of nocturnal mammals in Araucanía, correlating with güiñas' nocturnal activity. Camera trap studies confirm güiñas in southern Chile are predominantly nocturnal, aligning hunting with small mammal prey activity (Delibes‐Mateos et al. 2014; Hernández et al. 2015; Figueroa et al. 2018). Camera traps did not capture the adult bringing prey to the cavity, implying it leaves the kitten/cub alone while hunting and only nurses it upon return. Güiñas maximize prey encounters through extensive searching, like other forest‐dwelling felids (Freer 2004; Peckham 2023). Their consumption of nocturnal and diurnal prey aligns with their circadian rhythm (Dunstone et al. 2002; Sanderson et al. 2002; Delibes‐Mateos et al. 2014; Galuppo 2014; Hernández et al. 2015).

The period of birth and rearing of the kitten/cub in the observed den corresponds to that described by Dunstone et al. (2002) and Freer (2004), who each collected a juvenile male güiña. Based on size and dentition, they suggest that their births occurred between late October and early November. The adult was recorded breeding a single kitten/cub, and our camera trap data show a productivity of one young per female. Information on litter size or female productivity of güiña is scarce in the literature, though some articles indicate that litters can consist of 1–3 kittens/cubs (Iriarte 2008) or even up to 4 kittens/cubs (Peckham 2023). Other Leopardus species also show small litter sizes. For example, ocelots ( *Leopardus pardalis* ) and margay (*Leopardus weidii*) have 1 young per litter as the mode, while the closest species to güiña, the Geoffroy's cat ( *Leopardus geoffroyi* ), have 1–3 (Mittermeier and Wilson 2009). Although we did not collect data on the number of kittens/cubs at birth, our results suggest that the güiña may commonly raise only one young at a time. Moreover, despite published data on the duration of rearing outside the den not being available for this species, our observations of adults accompanied by offspring across multiple seasons suggest that the rearing period likely spans several months, as reported for ocelots and margay (Mansard 1997; Laack et al. 2005). This information is important for assessing the population status of the species and for future studies on reproductive behavior.

We suggest conducting systematic sampling of breeding sites to determine whether güiñas construct nests. The use of tree cavities should be considered in conservation management and monitoring plans for the species, particularly for monitoring the use of these structures and ensuring their preservation. We recommend additional studies to enhance our understanding of the breeding biology of the güiña in southern Chile. These studies could include evaluations of breeding habitat selection and the life‐history traits of this forest‐specialist feline species.

## Author Contributions

**Fernando J. Novoa:** conceptualization (equal), data curation (lead), formal analysis (lead), investigation (lead), methodology (equal), project administration (lead), writing – original draft (lead), writing – review and editing (equal). **Mariana Ayala:** investigation (equal), project administration (lead). **José Infante‐Varela:** investigation (equal), methodology (equal), supervision (equal), writing – review and editing (equal). **José Tomás Ibarra:** funding acquisition (lead), methodology (equal), project administration (lead), validation (equal), writing – review and editing (equal). **Jesús Díaz:** data curation (equal), investigation (equal), writing – review and editing (equal). **Tomás A. Altamirano:** funding acquisition (lead), methodology (equal), project administration (lead), validation (equal), writing – review and editing (equal). **Nicolás Gálvez:** conceptualization (equal), funding acquisition (lead), investigation (equal), methodology (equal), supervision (equal), validation (lead), writing – review and editing (equal).

## Conflicts of Interest

The authors declare no conflicts of interest.

## Supporting information


Data S1.

## Data Availability

All data are included in the main text of the paper. The complete dataset of tree‐cavity denning observations from this study is available as Supporting Information.
